# Imaging findings in a male pediatric patient by central precocious puberty: A case report

**DOI:** 10.1016/j.radcr.2025.09.065

**Published:** 2025-10-15

**Authors:** Raisa Mahmudah, Radinda Amalia Nur Hayati, Harry Galuh Nugraha, Ahmad Fitrah, Tasiya Ocvianty

**Affiliations:** Department of Radiology, Faculty of Medicine Padjadjaran University, Dr. Hasan Sadikin Hospital Bandung, West Java, Indonesia

**Keywords:** Precocious puberty, Pituitary microadenoma

## Abstract

Precocious puberty (PP) refers to the early onset of secondary sexual characteristics, occurring before 8 years of age in females and 9 in males. This condition commonly results from a premature activation of the hypothalamic-pituitary-gonadal (HPG) axis, a phenomenon identified as central precocious puberty (CPP). Central precocious puberty (CPP) in females is predominantly idiopathic, whereas in males, it is mostly caused by hypothalamic-pituitary lesions. Diagnosis is established through comprehensive clinical assessment, encompassing a thorough anamnesis from both the patient and their caregivers, detailed physical examination, and Tanner staging conducted by a pediatric endocrinologist. Neuroimaging, particularly brain MRI, is employed to detect intracranial anomalies or pituitary pathology, with pituitary microadenomas being a commonly observed finding. This report presents the case of a 5-year-old male who exhibited signs of secondary sexual development, with testicular volume consistent with Tanner stage 5 based on laboratory evaluation. Assessment of skeletal maturation revealed a significantly advanced bone age, approximating that of a 11-year-old child. The patient underwent an ultrasound examination by the results of testicular volume greater than the normal value of his age. Due to the results of the examination, the patient underwent an MRI examination of the head to find the cause by the conclusion on MRI, namely pituitary microadenoma.

## Introduction

Precocious puberty (PP) is a developmental condition in pediatric patients, characterized by the premature emergence of secondary sexual traits occurring before 8 years of age in girls and before 9 years in boys [1]. The main physical features associated by gonadarche in boys are as follows: a) Testicular enlargement. b) Pubertal and penile enlargement. c) Growth spurt. d) Bone age. e) Voice breaking. f) Miscellaneous: acne, increased muscle mass [2].

PP is broadly classified into 2 types: central precocious puberty (CPP) and peripheral precocious puberty (PPP). CPP may occur idiopathically or be associated by underlying abnormalities of the central nervous system (CNS), including neoplasms, cysts, or hydrocephalus [3]. Pituitary adenomas are the most commonly observed lesions within the sellar region. In the diagnostic evaluation of precocious puberty (PP), brain magnetic resonance imaging (MRI) is routinely utilized to investigate potential structural anomalies, particularly within the pituitary gland. Pituitary microadenomas are among the recurrent findings detected during this imaging assessment.

This case discusses a boy by PP who was found to have central abnormalities on MRI examination.

## Case report

### Clinical history and laboratory findings

A 5-year-old male presented with signs of accelerated growth and pubertal progression, including rapid increase in stature, testicular enlargement, development of pubic hair, penile growth, growth spurts, voice deepening, facial acne, and emergence of facial hair—all of which had reportedly begun at the age of 3. The patient sought medical evaluation upon reaching 5 years of age. Neither the patient nor his parents had experienced seizures, headaches, cognitive or behavioral problems, head injuries, CNS infections, diplopia, vomiting, intolerance to cold, or sex hormone medications. There was no history of radiation, previous encephalitis, or significant family history. At the time of presentation, the boy's body mass index (BMI) was greater than the 95th percentile (BW: 31 kg BH: 131 cm = 1.31 m, BMI: 23) as shown on the CDC growth chart.

Laboratory evaluation revealed a markedly elevated serum testosterone level of 801 ng/dL, consistent with Tanner stage 5, while luteinizing hormone (LH) and follicle-stimulating hormone (FSH) levels were both suppressed (<0.1 mIU/mL). Levels of growth hormone, 17-hydroxyprogesterone, and thyroid function markers were within normal reference ranges.

### Radiology findings

**Fig. 1:**
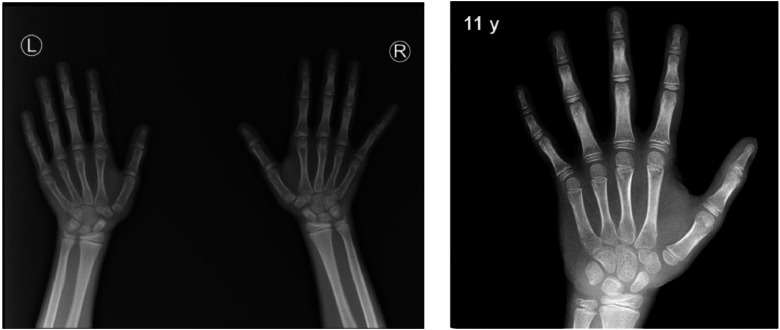
Bone age shown of approximately 11 years (Greulich and Pyle criteria).

**Fig. 2:**
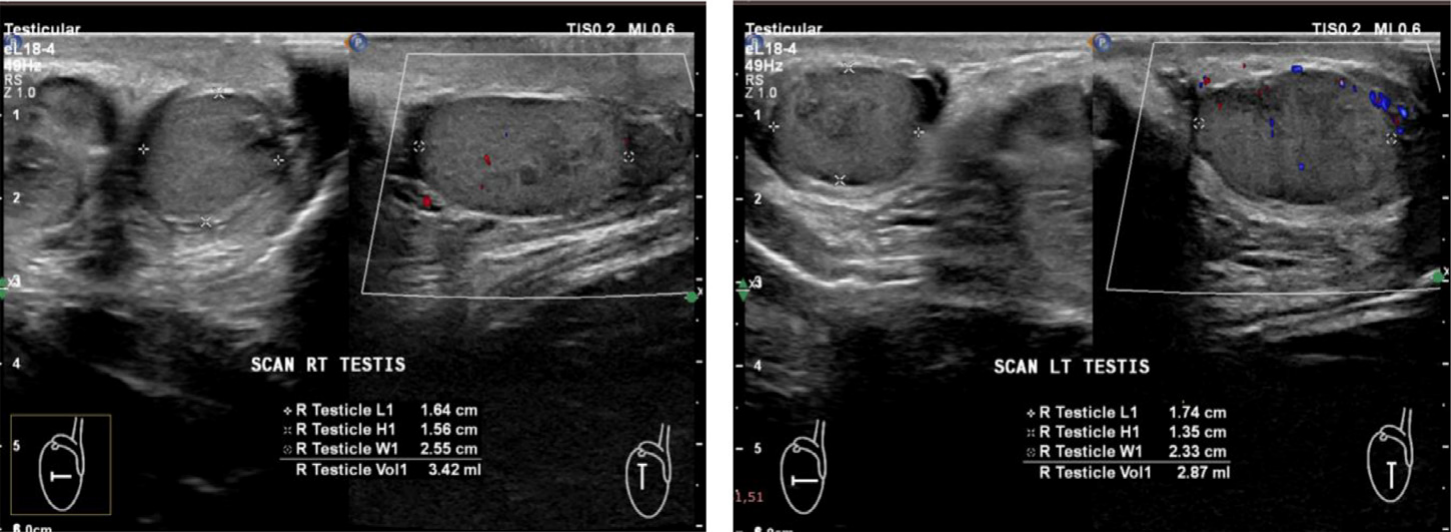
Testicular volume increased by approximately 3.42 mL in the right testicle and 2.87 mL in the left testicle.

Wrist radiographs showed bone age of about 11 years (Greulich and Pyle criteria) (Fig. 1). The testicular volume was seen to increase by approximately 3.42 mL in the right testis and 2.87 mL in the left testis (normal value for children aged 1-10 years is 0.7 ± 0.9 mL) during ultrasound examination (Fig. 2). From the results of laboratory examination as well as ultrasound and bone age, the patient was diagnosed by early puberty.

**Fig. 3:**
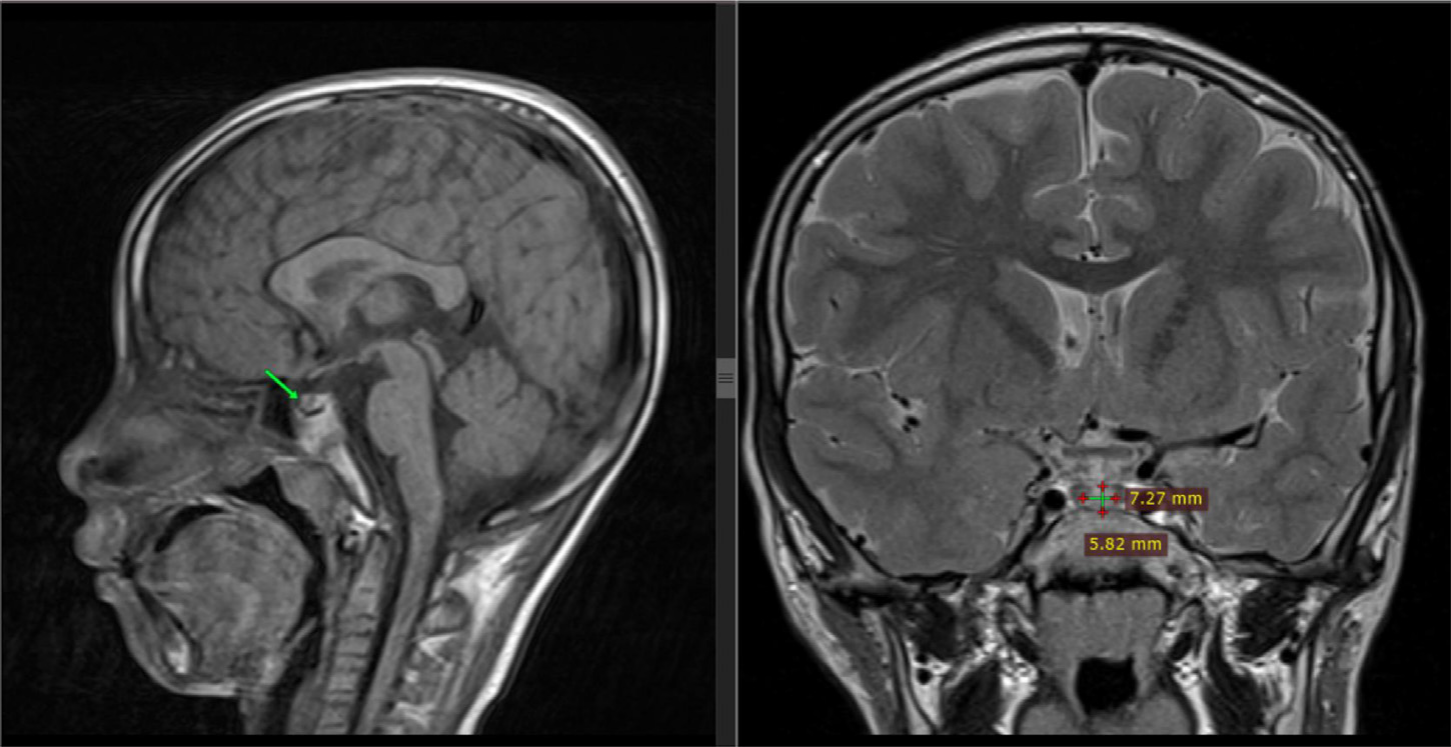
Lesion on anterior pituitary indicating suspected pituitary microadenoma.

Next, the cause was sought and the patient underwent Magnetic resonance imaging examination. MRI revealed a lesion on the anterior pituitary measuring approximately 0.72 × 0.58 cm which gave hyperintense signal intensity changes on T1W1 and gave enhancement on post contrast scanning which indicated a suspected pituitary microadenoma (Fig. 3).

## Discussion

PP is an endocrine condition observed in pediatric populations, marked by the premature emergence of secondary sexual characteristics significantly earlier than typical developmental timelines. Clinically, it is diagnosed when pubertal changes appear before 8 years of age in girls and before 9 in boys. In male patients, early gonadal activation (gonadarche) is primarily evidenced by testicular hypertrophy, the development of pubic hair, penile enlargement, accelerated linear growth, skeletal maturation beyond chronological age, and voice deepening. Additional signs may include the onset of acne and increased muscular development.

PP is broadly categorized into 2 primary forms: CPP and peripheral precocious puberty (PPP). CPP, the gonadotropin-dependent variant, arises from the early activation of the hypothalamic–pituitary–gonadal (HPG) axis. In CPP, the hypothalamus initiates premature secretion of gonadotropin-releasing hormone (GnRH), which in turn stimulates the anterior pituitary to release LH and FSH. This hormonal cascade accelerates gonadal maturation and leads to the early emergence of secondary sexual characteristics [4]. The diagnosis of CPP is confirmed through an in-depth clinical assessment comprising a detailed medical history obtained from both the patient and their caregivers, along with a comprehensive physical examination performed by a pediatric endocrinologist. Pubertal progression is evaluated using Tanner staging, while anthropometric parameters—such as height and BMI—are measured and interpreted against percentile benchmarks established by the Centers for Disease Control and Prevention (CDC) growth charts.

In this case, a 5-year-old boy presented by rapid height gain, testicular volume enlargement, pubic hair growth, penile enlargement, growth spurt, deepening of voice, facial acne, and facial hair growth that started at the age of 3 years. Neither he nor his parents reported any seizures, headaches, cognitive or behavioral problems, head injuries, CNS infections, diplopia, vomiting, intolerance to cold, or sex hormone medications.

Upon clinical presentation, the patient’s BMI measured 23, placing him above the 95th percentile according to CDC growth standards, with a recorded body weight of 31 kg and height of 131 cm. Laboratory analyses indicated a markedly elevated serum testosterone level of 801 ng/dL, consistent with Tanner stage 5 pubertal development. In contrast, both LH and FSH concentrations were notably suppressed (<0.1 mIU/mL). Further endocrine evaluations—including assays for growth hormone, 17-hydroxyprogesterone, and thyroid function—demonstrated values within normal physiological limits.

Radiographic imaging of the left hand and wrist was undertaken to evaluate skeletal maturation, accompanied by a testicular ultrasound examination. Bone age was determined by a pediatric endocrinologist utilizing the Greulich and Pyle atlas as the established reference framework [5]. The wrist radiograph showed a bone age of approximately 11 years (Greulich and Pyle criteria). Testicular volume was seen to increase by approximately 3.42 mL in the right testicle and 2.87 mL in the left testicle (Normal value of 1-10 years of age is 0.7 ± 0.9 mL) during ultrasonographic examination.

CPP may arise idiopathically or in association with underlying CNS abnormalities, including neoplasms, cysts, or hydrocephalus. The prevalence of incidental intracranial lesions identified in children diagnosed with CPP varies across studies and appears to differ by gender. In females, CPP is predominantly idiopathic, while in males, it is more frequently associated with hypothalamic–pituitary abnormalities. As part of the diagnostic workup for PP, brain magnetic resonance imaging (MRI) is routinely performed to detect potential structural abnormalities or pituitary lesions. Among the commonly observed findings are pituitary microadenomas, which are defined as pituitary lesions measuring less than 1 cm in diameter and not attributable to non-adenomatous etiologies such as cysts or infarcts [6].

The patient was examined Magnetic resonance imaging revealed a lesion in the anterior pituitary measuring lk. 0.72 × 0.58cm which gave hyperintense signal intensity changes at T1W1 and gave enhancement on post contrast scanning which indicated the presence of Suspected Pituitary Microadenoma. So it is known that the patient's PP is due to a lesion in the CNS.

At the time of this report, the patient has not returned for follow-up and has not received any further treatment

## Conclusion

This case shows a picture of CPP which on MRI examination found a pituitary microadenoma that disrupts the production of growth hormone and causes signs of PP in patients. Precocious puberty in patients is obtained from the results of a physical examination in the growth of pubic hair, on ultrasound and laboratories there is an increase in testicular volume (Tanner stage 5), and in bone age there is a mismatch in the description of the bones by his current age.

## Patient consent

**Confidentiality:** All personal data obtained is confidential which is guaranteed by the author. The author will not publish personal information of subjects that is not related to research interests.

**Participation:** Subject participation in this study is voluntary. Subject will not be compensated for the participation in this study. Subject may withdraw her participation at any time. Subject may also choose not to participate in this study.

**Risks:** Subject will not be compensated for her participation. However, subject will have the satisfaction of contributing to our knowledge and understanding of microadenoma in child with central precocious puberty. If Subject would like to receive one, Author will provide Subject with a preliminary report of my findings.

**Informed consent:** Subjects voluntarily participate in this study. Subject agree that Subject have given the opportunity to ask questions and have them answered to Subject satisfaction. Subject have received a copy of this consent form signed by the researcher.
